# Significant Productivity Improvement of the Baculovirus Expression Vector System by Engineering a Novel Expression Cassette

**DOI:** 10.1371/journal.pone.0096562

**Published:** 2014-05-13

**Authors:** Silvia Gómez-Sebastián, Javier López-Vidal, José M. Escribano

**Affiliations:** 1 Alternative Gene Expression S.L. (ALGENEX), Madrid, Spain; 2 Department of Biotechnology, Instituto Nacional de Investigación y Tecnología Agraria y Alimentaria (INIA), Madrid, Spain; Wuhan Bioengineering Institute, China

## Abstract

Here we describe the development of a baculovirus vector expression cassette containing rearranged baculovirus-derived genetic regulatory elements. This newly designed expression cassette conferred significant production improvements to the baculovirus expression vector system (BEVS), including prolonged cell integrity after infection, improved protein integrity, and around 4-fold increase in recombinant protein production yields in insect cells with respect to a standard baculovirus vector. The expression cassette consisted of a cDNA encoding for the baculovirus transactivation factors IE1 and IE0, expressed under the control of the *polyhedrin* promoter, and a homologous repeated transcription enhancer sequence operatively *cis*-linked to the *p10* promoter or to chimeric promoters containing *p10*. The prolonged cell integrity observed in cells infected by baculoviruses harbouring the novel expression cassette reduced the characteristic proteolysis and aberrant forms frequently found in baculovirus-derived recombinant proteins. The new expression cassette developed here has the potential to significantly improve the productivity of the BEVS.

## Introduction

The baculovirus expression vector system (BEVS) is one of the most powerful, robust, and versatile eukaryotic expression systems available. Given its development speed and versatility for the expression of a wide range of protein families, the BEVS offers multiple advantages for protein production in a variety of applications. The baculovirus vector most commonly used in industry and research laboratories for recombinant protein production is based on *Autographa californica* multinuclear polyhedrosis virus (*Ac*MNPV) with *Spodoptera frugiperda* 9 (*Sf*9) or 21 (*Sf*21) insect cells, *Trichoplusia ni* (*T. ni*)-derived High Five (Hi-5™) cells, and also whole *T. ni* insect larvae as suitable expression hosts (for review see [1], [2], [3]). Since the development of the BEVS in the ’80s [4], thousands of recombinant proteins, ranging from cytosolic enzymes to membrane-bound proteins, have been successfully produced in baculovirus-infected insect cells. However, like any other expression system, the BEVS has a number of bottlenecks, one of which regards the expression yields obtained in insect cells, which are significantly lower than those achieved with the most productive mammalian cells. While common expression yields in optimised transformed fed-batch mammalian cells cultured in a bioreactor may reach grams of recombinant protein per L, in insect cells infected by recombinant baculoviruses the yield rarely exceeds 50 to 100 mg per L. This relatively low expression capacity can be compensated in the BEVS by the short development times and lower costs associated with a specific product. This implies that for the production of a recombinant protein with market needs not exceeding 5 to 10 Kg per year (i.e. subunit vaccines), the BEVS is currently one of the best alternatives [5]. In fact, most licensed products obtained in insect cells correspond to vaccines and not to products with a high production demand such as therapeutic antibodies [6]. However, the yield is not the only bottleneck of the BEVS. A marked proteolysis of recombinant proteins during baculovirus-based production is frequently encountered. This observation is due, in part, to the cytopathogenic effects of the baculovirus vectors in insect cells during infection [7], [8], [9].

Considerable research effort has been channelled into increasing the productivity of the BEVS [2]. A variety of transfer vectors encoding resident fusion proteins reported to improve protein expression are now available for the construction of recombinant baculoviruses. These include maltose binding protein [10], glutathione S transferase [11], SUMO [12] and KDEL retention signal [13]. The deletion of other non-essential virus genes encoding for proteins such as p26, p10 and p74 also offer advantages for productivity [14]. Other attempts to improve the stability of the proteins expressed have focused on two genes in the baculovirus genome that are not essential for virus growth in cell culture, namely *chiA* (chitinase) [14], [15] and *cath* (cathepsin) [16], [17]. Finally, other approaches to improve baculovirus vectors involve the generation of non-lytic BEVS by random mutagenesis of viral genomes [18] and the incorporation of foreign genes (vankyrin genes) from the insect virus *Campoletis sonorensis ichnovirus* [19].

The acceleration of recombinant protein expression, before the machinery of insect cells is severely impaired by the infection with the virus vector, would greatly contribute to improving the productivity of baculovirus vectors. Late expression, driven by the conventional strong virus promoters of *polyhedrin* (*polh*) or *p10* genes, has serious disadvantages for post-translational modifications of the foreign protein. The apoptosis associated with baculovirus infection in insect cells is one of the most remarkable virus-induced cytopathogenic effects. Earlier baculovirus promoters have been characterised and used for heterologous protein production, but show reduced productivity in comparison to conventional very late promoters [20]. Additionally, the productivity of infected insect cells peaks before 72 h post-infection while declining dramatically thereafter. Long-term protein expression in the BEVS would enhance the productivity of this technology, especially for secreted proteins, which would accumulate in much larger amounts in the culture media. Therefore prolonging the survival of infected cells by inducing baculoviral resistance is another priority to address for the advancement of the BEVS platform.

Here we developed a baculovirus expression cassette containing various baculovirus genomic elements, such as transactivators (IE0 and IE1), an enhancer sequence (*hr1*), and a number of promoters or combinations of promoters interacting with each other and acting in a cascade. When introduced into the AcMNPV-based vector genome, this novel expression cassette conferred to the BEVS an increased cell viability and integrity late after infection and, most importantly, unprecedented early-late expression levels. Together, these properties increased the integrity of the recombinant protein, thus boosting its functionality.

## Methods and Materials

### Cell culture and viruses

*Trichoplusia ni* (High Five™, Hi-5™) and *Spodoptera frugiperda* (*Sf*21 and *Sf*9) cell lines were cultured at 27 °C in TNMFH medium (PAN Biotech GmbH, Germany) with 10% heat-inactivated fetal bovine serum (PAN Biotech GmbH) and gentamicin (50 µg/ml) (PAN Biotech GmbH). Cell density and viability were assessed by Trypan blue staining. Cell viability was calculated on the basis of the percentage of living cells with respect to the total number of cells at various times post-infection. The recombinant baculoviruses (rBacs) were obtained by generating the bacmids by means of the pFastBac1 or pFastBacDual vectors for use with the Bac-To-Bac baculovirus expression system (Invitrogen, Life Technologies). Bacmids were transfected into *Sf21* cells using Cellfectin®II Reagent (Invitrogen, Life Technologies) and following the manufacturer's instructions. In addition, the polhAc-ie-01/*hr1p6.9p10GFP* rBac was generated using the OET flashBAC™ system and following the manufacturer's instructions. The resulting rBacs were then passaged twice and titrated in duplicate by plaque assays in 6-well plates. Titers were expressed as plaque-forming units (PFU).

Cells were cultured in monolayer in 6-well plates and infected with the different viruses at a density of 5×10^5^ cells/well in the case of Hi-5 and at a density of 10^6^ cells/well for *Sf21*. The *Sf9* cells, cultured in suspension, were infected in spinner flasks (80 ml of culture media) at a cell density of 2×10^6^ cells/ml. The viability of the cells at the moment of infection was >95% in monolayer and >99% in suspension. Infected cells were examined under a Leica DMIL inverted microscope.

### Plasmid constructions

**Figure 1:**
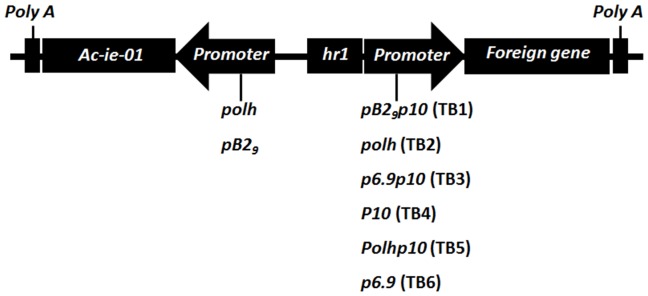
Schematic representation of genetic components of the baculovirus expression cassette (TB) developed.

Plasmid *polhAc-ie-01* was constructed by cloning a synthetic (GenScript) cDNA *Ac-ie-01* sequence (*Ac*MNPV complete genome, GenBank accession n° NC_001623) into Bam*H*I and XbaI sites of pFastBac1. The GFP gene was ligated into the Eco*R*I *and* SphI sites of pFastBac1 and into the SphI site of pFastBacDual vectors to generate *polhGFP* and *p10GFP*, respectively. To create the plasmid *p6.9GFP*, the promoter sequence was synthesised (GenScript) and cloned into Bst*Z17*I and XhoI sites of plasmid *polhGFP*, where *polh* was substituted by the *p6.9* sequence. Plasmids *hr1polh, hr1p10 and hr1p6.9* were constructed with synthesised DNAs (GenScript) and cloned into the BstZ17I and *Xba*I sites of pFastBacDual and the GFP gene was inserted into the HindIII site. Expression cassettes *TB1* to *TB6* were generated following several steps: the DNA sequences *hr1pB2_9_p10 (TB1), hr1polh (TB2), hr1p6.9p10* (*TB3*), *hr1p10 (TB4), hr1polhp10 (TB5) and hr1p6.9p10* (*TB6*) were synthesised (GenScript) and cloned into the BstZ17I and XbaI sites of pFastBacDual, while the *polhAc-ie-01* sequence was inserted into BstZ17I and PvuII of the same vector. The GFP gene was inserted into the HindIII site. In the case of the DNA coding sequence of a single domain antibody against the rotavirus A VP6 protein fused to the GFP (*VHHGFP*), it was synthesised with a 6-His tag in the C-terminal site and cloned into pFastBac1 using the BamHI and HindIII restriction sites. The *TB3VHHGFP* was generated as follows: *VHHGFP* was amplified by PCR to include the 5′-XhoI and 3′-NcoI flanking restriction sites and was inserted into the same sites in the *TB3* plasmid. Figure 1 shows a schematic representation of all different *TB* expression cassettes generated in the present work.

Additionally, the *Ac-ie-01* cDNA was also synthesised (GenScript) under the control of *pB2_9_* promoter [21]. The resulting construct (*pB2_9_Ac-ie-01*) was cloned into the BamHI and XbaI sites of *pFastBac1*.

### Determination of protein expression

*Sf*21 and *Sf*9 cell lines were infected with the rBacs at a multiplicity of infection (m.o.i.) of 5 or 0.1 PFU. Cell cultures were harvested at a range of times post-infection and sedimented by centrifugation. The supernatants were removed, and the pellets were resuspended in PBS pH 7.2 and disrupted by three cycles of freezing (−196°C) and thawing (37°C). Cellular debris was removed by centrifugation at 23,660×*g* for 5 min at 4°C. Protein concentrations were determined by the Bradford method using a protein assay kit (Bio Rad Laboratories). Fluorescence analysis of GFP from 20 µg of total soluble protein (TSP) was performed with a Tecan fluorescence plate reader (GENios) (excitation 485 nm, emission 535 nm). Thirty µg of TSP fractions from infected cells were resolved in 12% SDS-PAGE gels. Gels were stained with Coomassie blue or transferred to nitrocellulose membranes. Immune detection of GFP, VHHGFP, actin, and tubulin was performed by Western blot analysis using anti-GFP monoclonal antibody (Millipore), anti 6x-His monoclonal antibody (Clontech), anti-alpha tubulin monoclonal antibody (*Sigma*) and anti-actin polyclonal antibody produced in rabbit (Sigma), respectively. Immunocomplexes were revealed with anti-mouse IgG-horseradish peroxidase (HRP)-labelled conjugate (KPL, UK), diluted 1∶2,000 or by an anti-rabbit IgG-horseradish peroxidase (HRP)-labelled conjugate (KPL, UK), diluted 1∶2,000 as secondary antibody. Protein bands were detected using an ECL Western blotting detection system and analysed by the ChemiDoc XRS Gel Imaging System with Image Lab software (Bio-Rad). Recombinant GFP was measured by means of Pro260 chips (Bio-Rad) and analysed by capillary electrophoresis using the Experion system (Bio-Rad), following the manufacturer's instructions.

## Results

### The introduction in the baculovirus genome of a second copy of the transactivators IE1 and IE0, encoded by the *Ac-ie-01* cDNA, increases the viability of insect cells late after infection

Very late promoters (*polh* and *p10*) are commonly used to drive the expression of foreign genes in baculovirus vectors. This implies that the expression time-frame is very short as it must occur before the virus-induced cytopathic effects on cell viability. Since IE1 and its splice variant IE0 from *Ac*MNPV have been shown to affect viability of transfected insect cells, here we studied the effects on baculovirus infection of the introduction in the genome of a second copy of these regulatory factors expressed under the control of a strong promoter. For this purpose, we constructed a baculovirus expressing the *Ac-ie-01* cDNA, which encodes for IE1/IE0. This sequence was expressed under the control of *polh* promoter. As a control, a rBac expressing the GFP protein under the control of the same promoter was also used. Both baculoviruses were employed to infect *Sf*9 cells in suspension at a m.o.i. of 0.1.

**Figure 2:**
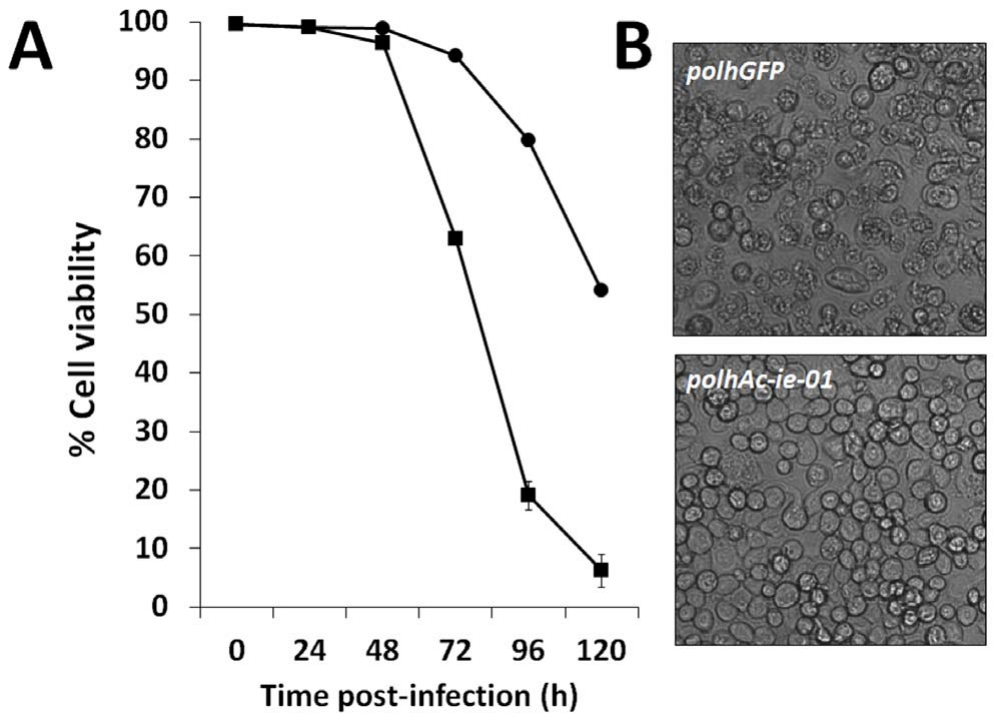
Increase of viability and integrity observed on insect cells infected with a baculovirus vector expressing the cDNA *Ac-ie-01*, encoding for the transactivators IE1/IE0, under the control of the *polh* promoter. A) Viability of cells infected (m.o.i. of 0.1) with control and *Ac-ie-01* recombinant baculovirus determined by Trypan blue staining at various times post-infection. B) Micrographs of Hi-5 cells cultured in monolayer and infected by the control or the above mentioned baculovirus at 96 hpi.

Analysis of cells infected by each baculovirus revealed that the virus vector incorporating the cDNA *Ac-ie-01* expressing extra copies of IE1/IE0 showed decreased cytopathic effects in cultures. Cell cultures infected by the two baculoviruses were analysed by Trypan blue staining to determine cell viability at various times post-infection. Interestingly, at very late times post-infection (96–120 h), cells infected by the virus incorporating the second copies of the transcriptional regulators showed an increase (50–60%) in cell viability with respect to cells infected by the control vector (Figure 2A). This observation suggests that a potential overexpression of these transcriptional regulators protects the cells from the cytopathic effects exerted by the baculoviruses, thus allowing long-term expression. The increased cell integrity results observed in *Sf*9 cells infected in suspension were also confirmed in Hi-5 cells cultured in monolayers (Figure 2B).

To determine the influence of the promoter on a baculovirus vector by the expression of the second copies of the IE1/IE0 transactivators, we generated another baculovirus expressing the *Ac-ie-01* cDNA under the control of a lower strength promoter derived from *Trichoplusia ni* lepidopteran insect and named *pB2_9_*. Similar results were observed with the new rBac (data not shown). Despite that we did not study the expression level of these factors obtained with different promoters in infected cells, it seems that promoter strength is not relevant for the functions of the second copies of IE1/IE0 when expressed in a baculovirus vector.

### Transactivators encoded by *Ac-ie-01* cDNA potentiate the *p10* baculovirus promoter cis-linked to the *hr1* enhancer sequence

Immediate early viral proteins from *Ac*MNPV encoded by *Ac-ie-01* cDNA (IE1/IE0) are potent transcriptional regulators of the baculovirus. Transactivation mediated by these proteins is enhanced by their binding as homodimers or heterodimers to the baculovirus homologous region (*hr*) sequences, which act as transcriptional enhancers. We analysed the effect of IE1/IE0 on several promoters or combination of promoters cis-linked or not to the enhancer sequence *hr1* in relation to the expression of a foreign protein (GFP). To demonstrate expression synergies of all of these genetic elements, various expression cassettes were prepared and used to generate the corresponding recombinant *Ac*MNPV rBacs. As control baculoviruses, three vectors were generated to express the GFP reporter gene under the control of *polh, p10* or *p6.9* promoters. The expression of GFP protein mediated by the baculoviruses was studied by fluorimetry at various times post-infection in *Sf*21 cells cultured in monolayers and infected at a m.o.i. of 5. GFP expression obtained with the *polh* promoter was considered the 100% value and incremental or detrimental percentages of fluorescence activity with respect to that obtained with this control virus were determined with all the rBacs assayed.

**Figure 3:**
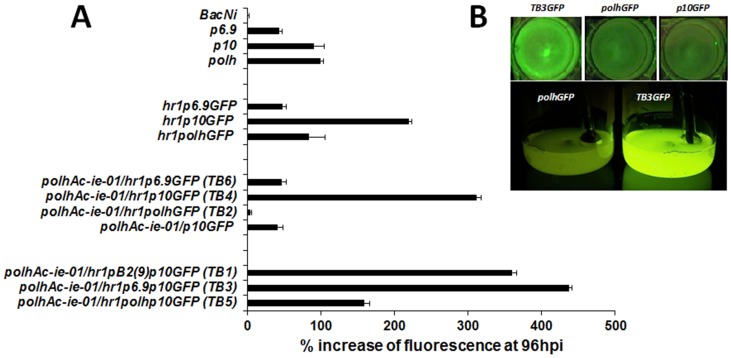
Recombinant GFP expression in *Sf*21 insect cells infected at a m.o.i. of 5 with rBacs carrying different expression regulatory genetic elements. A) Fluorimetric analysis expressed as arbitrary fluorescence units of cells infected at 96 hpi with the baculoviruses. Bars represent the media and standard deviation of three independent experiments. B) Visual fluorescence of *Sf*21 or *Sf*9 insect cells cultured in monolayer or suspension respectively and infected with the indicated baculoviruses at a m.o.i. of 5 (monolayer) or 0.1 (suspension) and observed under UV light.

The incorporation of the enhancer sequence *hr1* upstream to the three promoters analysed only increased significantly the strength of the *p10* promoter (Figure 3A). When the cDNA *Ac-ie-01* was incorporated into the expression cassette, similarly to what happened with the *hr1* enhancer, only improved the strength of the cassette carrying the *p10* promoter (*TB4*; Figure 3A). Surprisingly, other late promoters like *p6.9* (*TB6*) or *polh* (*TB2*) did not showed any increase in productivity. Moreover, the expression of GFP mediated by the *polh* promoter in the context of this expression cassette (*TB2*) dropped dramatically (Figure 3A). To clarify the synergistic effects of transcriptional regulators and the enhancer sequence *hr1* in the GFP expression levels obtained with *TB4*, we generated a baculovirus genetically modified with an expression cassette identical to *TB4* but lacking the enhancer sequence. With this baculovirus we aimed to demonstrate if transactivators were able to increase productivity per se or need the concurrence of the enhancer sequence. Interestingly, the GFP expression levels obtained with this virus were lower than that reached with a conventional baculovirus expressing the protein under the control of *p10* promoter alone (Figure 3A). This result clearly demonstrates that the overexpression of GFP mediated by *TB4* is the resulting synergistic interaction between the transactivators and the enhancer sequence.

Finally, we tested the synergistic effects on expression levels of combining the *p10* promoter with the other two promoters used in the present study (*p6.9* and *polh*) and with an additional *Trichoplusia ni*-derived promoter previously characterized in our laboratory, the *pB2_9_* (*TB1*). The resulting baculoviruses expressed higher levels than the reference virus used (*polhGFP*). However, the construct combining the promoters *p6.9* and *p10* (*TB3*) resulted in the most productive baculovirus expression cassette, which was able to increase productivities in about 4.5 times with respect to the control *polhGFP* expression cassette (Figure 3A). In conclusion, the highest incremental values with respect to the standard *polhGFP* were achieved with the expression cassette configuration *TB3*, followed in order by *TB1*, *TB4* and *TB5* (Figure 3A).

Differences in the visual fluorescence induced by a baculovirus harbouring *TB3* with respect to that induced by conventional baculoviruses using *polh* or *p10* promoters were very evident in infected cell cultures (in monolayer and suspension) under UV illumination (Figure 3B).

**Figure 4:**
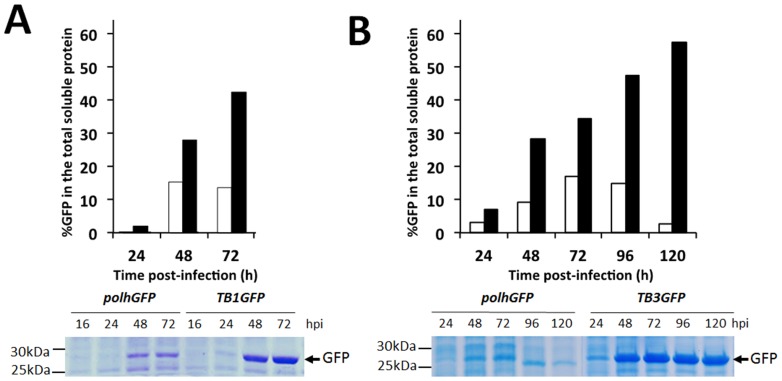
Production of GFP recombinant protein by insect cells infected by recombinant baculoviruses expressing the protein under the control of the TB1 cassette or the *polh* promoter, as determined by capillary electrophoresis using the Experion system. A) Comparison of GFP productivity (percentage of total soluble protein from cell extracts) in *Sf*9 cells cultured in monolayer and infected at a m.o.i. of 5 with a baculovirus expressing the reporter protein under the control of *TB1* expression cassette (black) or under the *polh* promoter (white). B) Comparison of GFP productivity (percentage of total soluble protein from cell extracts) in *Sf*21 cells cultured in suspension and infected at a m.o.i. of 0.1 with a baculovirus expressing the reporter protein under the control of the *TB3* expression cassette (black) or the *polh* promoter (white). Coomassie blue staining of SDS-PAGE gels resolving the proteins in the cell extracts in both experimental conditions are also shown in panels A and B. In both cases, the GFP band in cells infected by the baculovirus carrying the *TB* cassettes was more abundant than that expressed by the conventional baculovirus, thereby evidencing higher production yields and confirming the quantification results obtained by other methods.

A more precise quantification of the recombinant GFP was performed by capillary electrophoresis using the Experion system (Bio-Rad). This analysis was done with the TB1 cassette and was compared with the control (*polhGFP*) baculovirus. In extracts from *Sf*21 cells cultured in monolayer and infected with the baculovirus carrying *TB1* at a m.o.i. of 5, we achieved percentages of recombinant protein of about 40% of the total cell soluble protein fraction, while by using the conventional *polh* promoter we reached percentages not higher than 15% (Figure 4A). In cells infected in suspension with a *TB3*-modified baculovirus at a m.o.i. of 0.1, the differences were even higher, reaching 55% of the total soluble cell protein at 120 h post-infection, against the maximum productivity peak of 17% obtained with the reference virus (Figure 4B). Cell extracts analysed by SDS-PAGE and stained with Coomassie blue revealed the differences in GFP expression between the control baculovirus and that carrying the *TB1* or *TB3* expression cassettes, both in cells infected in a monolayer at a high m.o.i. (Figure 4A) and in those infected in suspension at a low m.o.i. (Figure 4B). The modified baculovirus produced more than 400 mg/L in suspension cultures, while the maximum peaks of productivity with the control baculovirus never exceeded 100 mg/L. Similar results were obtained with the *Trichoplusia ni*-derived Hi-5 cells, which attained 5-fold values with respect to a conventional baculovirus in cells cultured in a monolayer and infected at a m.o.i. of 5 (data not shown), as measured by fluorimetry.

### The regulatory elements contained in the novel expression cassette facilitate the post-translational processing of recombinant proteins and reduces protein proteolysis late after infection

Cellular integrity during baculovirus infection is crucial to ascertain the correct folding or any other post-translational modification of foreign proteins expressed by this system. The baculovirus strong promoters commonly used for research and production, such as *polh* and *p10*, express the foreign genes only at late times post-infection when cells are already affected by severe cytopathic effects and the viability of cultures has decreased. As described above, the incorporation of additional copies of the transcriptional regulators encoded by the cDNA *Ac-ie-01* in the baculovirus expression cassette protected the cells from the pathogenic effects of the infection, allowing a wide temporal window for recombinant protein production in cells remaining fully viable.

**Figure 5:**
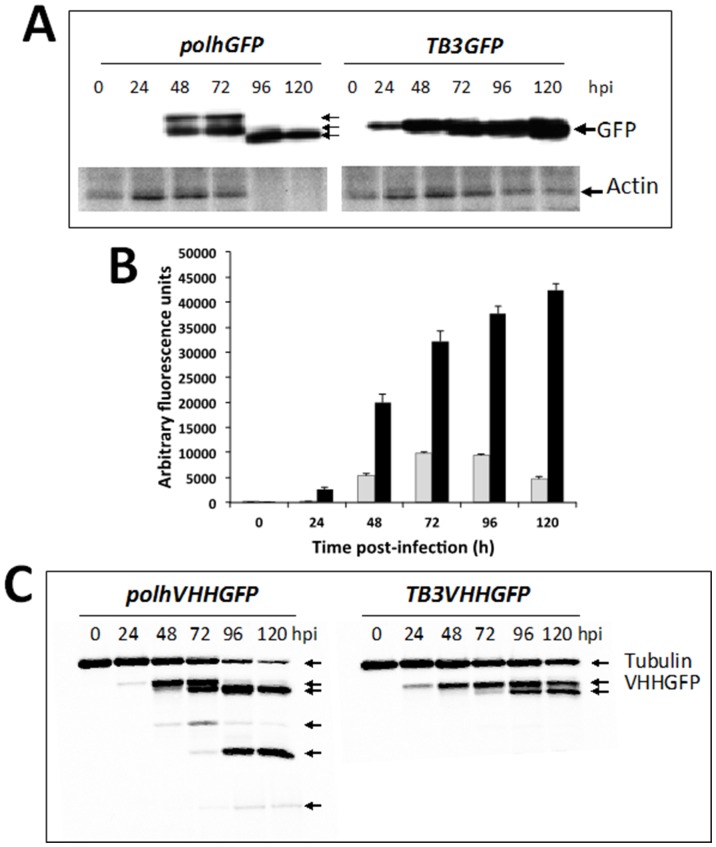
Influence of genetic regulatory elements in a *TB* expression cassette on the conformation, proteolysis and functionality of recombinant proteins expressed by a baculovirus vector. A) Analysis by SDS-PAGE and Western blot with an anti-GFP antibody of cell extracts recovered at various times post-infection with a recombinant baculovirus expressing the GFP protein under the control of the *polh* promoter or the *TB3* expression cassette. Different reacting protein bands indicated by arrows can be observed at each time point post-infection with the conventional baculovirus while only a reactive band of the expected molecular weight for GFP is observed in the cell extracts from cells infected with the virus engineered with the *TB3* cassette. Actin integrity in the different cell extracts was studied by Western blot using a specific antiserum, showing differences of reactivity among the baculoviruses analysed at late times post-infection. B) Fluorescence activity of cell extracts recovered at different times post-infection with the two analysed baculoviruses and determined by fluorimetry. While the values increase over time in the case of *TB3*-modified baculovirus, the values peaked at 72 hpi in extracts from cells infected by the conventional baculovirus, decreasing significantly at later times post-infection. C) Analysis by SDS-PAGE and Western blot with anti-His tag and anti-tubulin antibodies simultaneously of cell extracts recovered at different times post-infection with a recombinant baculovirus expressing a single-domain antibody fused to GFP protein (VHHGFP) under the control of *polh* promoter or under the *TB3* expression cassette. A number of degradation bands indicated by arrows were clearly observed in cells infected with the conventional baculovirus expressing the fusion protein after 48 hpi, in contrast to that observed when the fusion protein was expressed under the control of *TB3* expression cassette. Tubulin integrity determined by Western blot analysis in the different cell extracts showed differences of reactivity among the baculoviruses analysed at late times post-infection.

We studied the relevance of the prolonged cell viability conferred to the baculovirus vector by the novel expression cassette in relation to the processing of recombinant proteins, with a special focus on proteolysis. For this purpose, a conventional baculovirus expressing the reporter protein GFP under the control of the *polh* promoter and a baculovirus incorporating *TB3* and also expressing the GFP protein were used to infect *Sf*9 insect cells in suspension at a m.o.i. of 0.1. The cells were analysed at different times after infection by Western blot using an anti-GFP monoclonal antibody (mab 2515; Millipore™). Interestingly, GFP expressed by a conventional baculovirus showed several reactive bands at 48 and 72 h post-infection (suggesting differences in the processing of the protein) and, at later times, a band with a reduced molecular weight (lower than predicted), suggesting degradation (Figure 5A). In contrast, when GFP expression was mediated by *TB3*, only one GFP band showing the expected molecular weight of this protein was observed at all the post-infection times analysed (Figure 5A). In this experiment, we also confirmed that TB3 conferred to the baculovirus vector long-term expression, since the GFP production by this modified vector was not significantly reduced at a very late time post-infection (120 h).

In parallel, the integrity of an endogenous cellular protein, indicative of cell wholeness, was also measured at various times post-infection. For this purpose, we used Western blot analysis with a specific antiserum to study cellular actin. After 72 h post-infection, the actin band decreased or even was not detected in protein extracts from cells infected by the conventional baculovirus. Consistent with the cellular protection induced by the recombinant DNA elements in *TB3*, cellular actin was not equally affected in cells infected by the engineered rBac carrying this expression cassette (Figure 5A).

The fluorescence activity of recombinant GFP expressed by a rBac reflects the level of expression of this protein and its correct conformation. The GFP expressed under the control of *TB3* maintained an increased functionality over infection times. In contrast, the fluorescence activity of the GFP expressed by a conventional baculovirus peaked at 72 h post-infection and decreased thereafter, in parallel to actin degradation and decreased cell viability (Figure 5B).

In order to corroborate the potential reduction of recombinant protein proteolysis induced by baculoviruses harbouring the novel expression cassette during infection, we generated 2 new baculoviruses expressing a single domain antibody specific for the VP6 protein of rotavirus A named 3B2, transcriptionally fused to the GFP reporter protein (VHHGFP). This fusion antibody was expressed under the control of the *polh* promoter or in the context of *TB3*. *Sf*9 insect cells were infected with each baculovirus in suspension at a m.o.i. of 0.1, and cell extracts taken at a range of times post-infection were resolved in SDS-PAGE gels and analysed by Western blot using an anti-GFP antibody. We observed a number of degraded reacting bands of the recombinant fusion protein produced under the control of the *polh* promoter from 48 h post-infection. These degradation bands were more remarkable after 96 h post-infection (Figure 5C). In contrast, in cells infected with the baculovirus carrying *TB3*, minor levels of degradation were observed even at very late times after infection (Figure 5C). Cell integrity was measured in this case by the constitutive cellular protein tubulin, which was also observed to be better preserved in the *TB3* genetically modified baculovirus than in cells infected by the conventional one (Figure 5C).

### The *TB* expression cassettes are fully compatible with transposition- and homologous recombination-based technologies used to obtain recombinant baculoviruses

**Figure 6:**
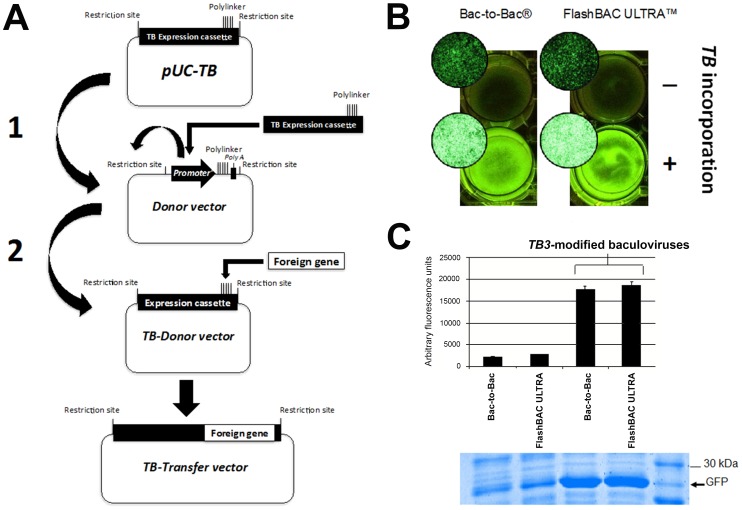
Analysis of the compatibility of *TB* cassettes with commercial site-specific transposition and homologous recombination technologies commonly used to generate recombinant baculoviruses. A) General strategy followed to obtain the recombinant baculoviruses modified by a *TB* expression cassette using the Bac-to-Bac and FlashBAC ULTRA commercial systems. Only one additional cloning step is required to introduce the *TB* expression cassettes into any commercial methodology used to produce a recombinant baculovirus. B) Visual fluorescence of *Sf*21 insect cells infected at 72 hpi by conventional or *TB3*-modified baculoviruses under UV illumination. This panel shows the cells both macroscopically and in micrographs obtained by a fluorescence inverted microscope. Fluorescence intensities were highly evident when the baculoviruses expressed the GFP protein under the control of *TB3*. These differences in fluorescence intensity were not significantly different between the two technologies used to generate the recombinant baculoviruses. C) Quantification by fluorimetry and Coomassie blue staining of SDS-PAGE gels of the GFP in cell extracts at 72 hpi with the different baculoviruses. Highly significant quantitative differences in fluorescence values and protein staining were observed between conventional baculoviruses and those genetically engineered with the *TB3* expression cassette.

We next sought to demonstrate the compatibility of the novel *TB* expression cassette with commercial technologies used to generate recombinant baculoviruses. For this purpose, we selected a system based on the use of site-specific transposition technology (Bac-to-Bac system, Invitrogen) and another one based on homologous recombination (FlashBAC ULTRA, Oxford Expression Technologies). The general strategy used to generate the two rBacs modified by *TB3* is shown in Figure 6A. The transgene (GFP) was cloned into a plasmid containing the *TB3* and the resulting construct was denominated donor vector. *TB3* containing the GFP gene was then subcloned into the corresponding commercial transfer vector. These transfer vectors were used to generate the rBacs following the supplier's instructions. Two additional control viruses were obtained by cloning the GFP encoding gene under the control of the *polh* promoter into the conventional unmodified commercial transfer vectors selected.

The resulting baculoviruses were used to infect *Sf*21 cells at a m.o.i. of 5, and the expression of GFP by the *TB3*-modified baculoviruses was compared by fluorescence microscope observation to that of the corresponding conventional ones. GFP expression was much higher in the former (Figure 6B). Quantification of GFP activity in cell extracts by fluorimetry and Coomassie blue staining of cell extracts resolved in SDS-PAGE gels corroborated the expression differences observed (Figure 6C). In conclusion, we confirm that the *TB* expression cassettes developed here, and in particular the most productive one, *TB3*, is fully compatible with technologies currently used to produce rBacs.

## Discussion

Two of the main reasons for using the BEVS mainly for research purposes but not for commercial production are the relatively low protein yields compared to other advanced production methodologies and the frequently found recombinant protein proteolysis in infected insect cells. Here we undertook a systematic study to determine potential baculovirus-derived regulatory elements acting in *cis* and *trans* with the potential to improve the production capacity of baculovirus vectors in insect cells. By combining certain virus-derived regulatory genetic elements in a specific rearrangement, the resulting expression cassettes significantly reduced some of the above-mentioned limitations of baculovirus vectors.

Two of these elements were the transactivating proteins IE0 and IE1 from the *Autographa californica* multiple nucleopolyhedrovirus (*Ac*MNPV). IE1, the widely studied product of the immediate early gene 1 (*ie1*), is a multifunctional protein of 66.9 kDa [22] involved in the regulation of the viral cycle through its capacity to differentially transactivate early and late viral genes [23], [24], [25] and to participate in the replication of the viral genome [26], [27]. This DNA replication activity is independent of the transcriptional activity of IE1 [28]. This protein colocalizes with viral DNA and essential viral DNA synthesis proteins within nuclear replication centres of infected cells [27], [29], [30]. When tested in transfected cells, IE1 participates in combination with virus-encoded DNA helicase P143, primase factors LEF-1 and LEF-2, single-stranded DNA-binding protein LEF-3, and DNA polymerase (DNA Pol) to replicate *hr*-containing DNA plasmids [31], [32]. The functional organisation of the baculovirus IE1 resembles that of transcriptional regulators of diverse vertebrate viruses and is highly conserved among members of *Baculoviridae*. IE1 transactivates its own promoter and that of the suppressor of apoptosis p35 and down-regulates the *ie0* promoter [32], [33].

Much less information is available on IE0, a 74-kDa protein identical to IE1 except for an additional 54 amino acid residues at its N-terminus, and thus with the same structural motifs as IE1. IE0 is the product of the immediate early gene 0 (*ie0*), which results from the splicing of exon 0 (38 amino acids) to the 5′ end of the untranslated leader of the *ie1* mRNA (16 aa) and the entire 582 aa of IE1 [34], [35], [36]. Also, IE1 is translated from *ie0* mRNA from its internal *ie1* start codon. Elimination of the *ie1* start AUG codon by mutating it to GCG prevents the translation of IE1 without affecting the function of IE0 [34]. IE0 is involved in the transactivation of *ie1* and *polh* promoters of *Ac*MNPV [34], [36] and up-regulates the expression of insect and mammalian promoters [24]. This protein localizes to the cell nucleus [37]. The role of IE0 in the *Ac*MNPV infectious cycle is unclear. In contrast to IE1, IE0 is expressed early in infection, peaking prior to DNA replication and declining late in infection [34], [38], [39], [40]. Moreover, it has been proposed that IE0 binds to viral DNA and that it interacts with IE1 in modulating the viral infection [38], [39], [40], [41]. Selective ablation of IE0 by RNAi results in delayed synthesis and lower steady-state expression of IE1 [42] and selective ablation of *ie1/ie0* blocks virus DNA synthesis and late gene expression in permissive *Spodoptera frugiperda* cells [42].

The IE1 protein has been shown to be necessary but not sufficient for expression of late and very late promoters, although it is sufficient for the expression of early promoters [22], [23], [24], [43]. The latter observation is consistent with previous studies using transient expression assays, which revealed the involvement of IE1 in determining the expression of other early promoters [43], [44], [45]. Given this observation, its requirement for the activation of late promoters may be indirect, involving the expression of other early transregulators or gene products that participate in DNA replication.

The incorporation of additional copies of these factors and expressing them under the control of a strong promoter (*polh*), probably above virus endogenous levels, in a baculovirus vector increased cell survival late after baculovirus infection. This property is extremely important in a baculovirus-based production system. Depletion of IE1 and IE0 abolished early expression of the caspase inhibitor gene p35, which prevents virus-induced apoptosis [42]. As IE1 transactivates the promoter of p35, the suppressor of apoptosis, the potential overexpression of this inhibitor may explain the observed increase in cell viability at late times post-infection. Expression of only IE0 has been also previously described to prolong the replication cell cycle of the virus, allowing cells live for longer periods of time after infection [40]. However, more research is required to account for the contradiction that IE1 is required for the baculovirus early replication events that trigger apoptosis in permissive and non-permissive cells [42], [46].

The preservation of an intact cell machinery improves recombinant protein conformation and reduces protein degradation by proteolysis caused by the release of cellular proteases in response to the virus. The activities of cellular proteases are often regulated by the metabolic state and oxidative stress of the cells. Heat shock and amino acid starvation induce the formation of various stress proteins, including proteases, that may have a significant impact on product quality. Here we observed evident differences in protein conformation and proteolysis when a TB cassette was used in 2 recombinant proteins analysed (Figure 5). The recombinant proteins expressed under the control of *polh* promoter presented aberrant forms of higher molecular weight than those predicted as well as proteolytic bands. In contrast, the same proteins expressed in the novel expression cassette described here showed the same molecular weight at all times post-infection. The functionality of the GFP expressed by the different baculoviruses, measured by fluorimetry, correlated with the protein integrity observed (Figure 5). Similar results were obtained with the chimeric single-domain fluorescent antibody in experiments of binding to its target protein [47], [48], [49] (VP6 protein from rotavirus A) in an ELISA test (data not shown), thereby indicating that the integrity of the protein also correlated with antibody functionality.

The other element used in the novel TB cassette was the baculovirus homologous region *hr1*, a transcription enhancer [22], [33], [41], [43], [50], [51], [52], [53]). In transfection assays, *cis* linkage of this and other *hr* elements to baculovirus promoters can boost IE1-mediated transactivation [45], [53], [54]. IE1 binds as a dimer to the 28-bp imperfect palindromic repeats (28-mers) that constitute the transcriptional enhancer activity of *hrs* and that are distributed throughout the circular DNA genome (134 kb) of *Ac*MNPV [33], [55], [56]. Transcriptional enhancement can be produced by homodimers of IE1 and IE0 and by heterodimers of IE1 and IE0 [41]. The combination of transactivators with the *hr1* sequence operatively linked with several promoters or combination of promoters in the TB cassette rendered varying levels of recombinant protein expression. Interestingly, our results indicate that in the context of a baculovirus vector, only when promoter *p10* was present in the expression cassette the expression detected was significantly increased with respect to that achieved by conventional cassettes using only the very late promoters *polh* or *p10* (Figure 3). Every element of the expression cassette (*Ac-ie-01* or *hr1*) by separate was insufficient to boost the productivity of the *TB* expression cassette (Figure 3), demonstrating that these elements are interconnected and act in cascade.

In conclusion, the *TB* baculovirus expression cassette described here may represent a significant improvement in the production of recombinant proteins by the BEVS. This cassette enhances cell viability and protein conformation, in addition to significantly increasing recombinant protein productivity. The simplicity of use of this expression cassette (Figure 6A) and its universality for the methods currently applied to produce rBacs (Figure 6B) render it highly suitable for the production of many different proteins. *TB* expression cassette has the potential to make insect cells more competitive with respect to other eukaryotic production systems.
